# Baseline blood count levels increase odds of cytopenia among CML patients in Kenya: a case control study

**DOI:** 10.1186/s12885-021-09162-z

**Published:** 2022-02-01

**Authors:** Angela McLigeyo, Jamilla Rajab, Peter Oyiro, Mohammed Ezzi, Yatich Bett, Matilda Ong’ondi, Andrew Odhiambo, Sitna Mwanzi, Nicholas Othieno-Abinya

**Affiliations:** 1 Department of Internal Medicine and therapeutics, Kenyatta University, Nairobi, Kenya; 2 Department of Human Pathology, School of Medicine, Unit of Hematology and Blood Transfusion, University of Nairobi, Nairobi, Kenya; 3 Hemato – Oncology Unit, Kenyatta National Hospital, Nairobi, Kenya; 4 Moi Teaching and Referral Hospital, Eldoret, Kenya; 5 Department of Clinical Medicine and Therapeutics, University of Nairobi, Nairobi, Kenya; 6 Aga Khan University Hospital, Nairobi, Kenya

**Keywords:** Imatinib, CML, Cytopenia, Philadelphia, BCR-ABL, Tyrosine Kinase

## Abstract

**Background:**

Imatinib is the gold standard for the treatment of all phases of Philadelphia positive Chronic Myeloid Leukemia (CML). During treatment, patients may develop cytopenia. We aimed to study the baseline characteristics and factors associated with cytopenia at a Nairobi Hospital.

**Methods:**

This was a retrospective case-control study of patients aged ≥18 years on follow-up at the Glivec International Patient Access Program (GIPAP) clinic from 2007 to 2015. The cases consisted of CML patients on imatinib who developed cytopenia. The controls were CML patients on imatinib who did not develop cytopenia. Baseline socio – demographic, clinical, hematologic, and molecular data were retrieved from patients’ files. Chi square or fishers’ exact tests were used to analyze for differences between cytopenia and no cytopenia. Binary logistic regressions were employed to identify relationships. Univariate and multivariate analyses were done to identify independent predictors of cytopenia. Odds ratios (OR) were presented including the 95% confidence intervals and respective *p* values.

**Results:**

A total of 201 patients were studied consisting of ninety-four (94) patients with cytopenia and 107 with no cytopenia. Among the entire population, males were 52, and 42% were aged 36–50 years. Sex, age, marital status, occupation and education level were similar between the cytopenia and no cytopenia groups.

Among the 201 patients, 70% had symptoms for > 12 months before diagnosis, 78.6% had B symptoms at baseline, 80% had a moderate splenomegaly at baseline.

Among patients with cytopenia, 40 and 37.4% developed cytopenia within 3 months and 3–6 months respectively after imatinib initiation. Baseline neutrophilia, neutropenia, anaemia, thrombocytosis, thrombocytopenia was found in 68, 11, 11, 23.5 and 11% respectively. Baseline hemoglobin, neutrophil and platelet level were significantly different between the cytopenia and the no cytopenia group. On univariable analysis, baseline anemia with hb < 7.9 g/dL (*p* = 0.002), neutropenia (*p* = 0.001), neutrophilia > 100,000/mm^3^ (*p* = 0.002) and thrombocytopenia (*p* = 0.001) increased the odds of developing cytopenia. On multivariable analysis, baseline anaemia (*p* value < 0.002), neutropenia (*p* value < 0.001), thrombocytopenia (*p* value, < 0.001) and thrombocytosis (p value, 0.033) increased the odds of developing cytopenia.

**Conclusion:**

Odds of cytopenia were higher in presence of baseline cytopenia and thrombocytosis. Clinicians should have a high index of suspicion for these patients.

**Supplementary Information:**

The online version contains supplementary material available at 10.1186/s12885-021-09162-z.

## Introduction

CML is a Philadelphia/BCR-ABL1-positive chronic neoplastic disease with an increase in blood granulocytes and bone marrow myeloid precursors, bone marrow hypercellularity composed of mature granulocytes and their precursors. Chronic myeloid leukaemia (CML) is due to a clonal disorder that cause granulocyte cell line proliferation [1]. It develops following a translocation that occurs reciprocally between two somatic chromosomes, t (9:22) [2].

The fusion protein resulting from this translocation, the BCR-ABL1, is a tyrosine kinase which acts independently of any stimulation and results in the development of CML [3]. Tyrosine kinase inhibitors (TKI) block this kinase and in turn block signaling pathways involved in myeloid proliferation while stimulating apoptosis and cellular adhesion [4, 5]. CML is more common in adults than in children and has an excellent 5 year overall survival [6]. The disease is also common in the elderly and in men in the developed world [6].

CML may be diagnosed in any of the three phases. Chronic phase which is the initial phase of CML characterized by increased neutrophils with various early-stage granulocytic precursors; accelerated phase which is characterized by 10–19% blasts in the bone marrow or peripheral blood, genomic evolution, persistent or increasing abnormal blood counts despite TKI treatment, (leukocytosis (> 10 ×  109/L), thrombocytosis (> 1000 × 109/L), or thrombocytopenia (< 100 × 109/L) unrelated to therapy, 20% or more basophils and splenomegaly; blastic phase which is characterized by more than 20% blasts in blood or bone marrow or extramedullary blastic infiltration, genomic evolution, splenomegaly [7] Myelosuppression may develop during treatment of CML with imatinib, a TKI, especially in the setting of advanced disease [8]. In first line therapy, imatinib 400 mg daily induces more neutropenia than nilotinib and bosutinib and slightly less than dasatinib. A differential effect between TKIs on other lines is less clear, although imatinib appears to induce slightly less thrombocytopenia and anaemia than dasatinib [9]. A thorough assessment of relevant comorbidities is crucial for guiding drug selection. In practice, imatinib is considered to have the mildest side effect profile among the TKIs [10].

Available data reported that longer time from diagnosis to treatment, prior interferon or imatinib therapy, and a lower white blood cell count at the initiation of TKI therapy were associated with an increased risk of grade II to IV cytopenia [11]. Guillot et al. also reported that advanced disease, baseline low hemoglobin, history of interferon induced cytopenia and previous busulphan therapy were risk factors for cytopenia [12]. Mauro et al. found that increased percentage of bone marrow blasts, low hemoglobin level, a longer time from diagnosis to treatment and a history of cytopenia were risk factors for cytopenia [13].

Cytopenia is a recognized problem at the Glivec International Patients’ Assistance Program (GIPAP) clinic in Nairobi. Its presence may be associated with poorer response to treatment [14]. Understanding the factors associated with its development during imatinib therapy will enable clinicians to plan for its prevention and management. We aimed to study the sociodemographic as well as the clinical and laboratory characteristics associated with increased odds of development of cytopenia among CML patients on imatinib at the GIPAP clinic.

## Methods

### Study Design, Aim and Setting

This was a retrospective case-control study of patients, ≥ 18 years of age, on imatinib, enrolled between 2007 and 2015. All data was retrospective and all charts were reviewed for both populations over a 36-month period for blood parameters, which for our patient population, was tested every 1–3 months during clinic review visits. It aimed to analyze the sociodemographic as well as the clinical and laboratory characteristics associated with increased odds of development of cytopenia among CML patients on imatinib. The Max access program provides imatinib therapy for patients enrolled in the GIPAP (CML) clinic at the Nairobi Hospital. Cumulatively, the clinic has 1200 patients. An average of 150 patients attend the clinic bi-weekly. The age range of patients seen in the clinic is 6 years to 75 years. Males in the clinic are in similar proportion to females and almost 90% present in chronic phase. Patients who initiate treatment are compliant with treatment with adherence rates of approximately 80% [15].

### Study population

CML patients aged ≥18 years, attending GIPAP clinic from 2007 to 2015 and on imatinib 400 mg daily who developed cytopenia ≥ grade 2 were enrolled. The definitions of cytopenia are included in a publication by the same group of a study preceding this one [16]. Cytopenia was determined from the complete blood count (CBC) report of hemoglobin, neutrophil and platelet counts. Monocytopenia was defined as an abnormality in one parameter, bicytopenia as an abnormality in two parameters and pancytopenia as an abnormality three parameters based. Categorization of severity was based on the National Cancer Institute Common Terminology Criteria for Adverse Events v.3 (NCI CTAE v3) [17].

### Sample size

The estimated required sample size for cases and controls was 76 each using a simple approximation for calculating sample sizes for comparing independent proportions by Fleiss (1980) [18]. Consecutive sampling was used. The cases were patients with cytopenia matched for age, sex and calendar year of enrolment with the controls, who had no cytopenia. A control was sampled each time a case was found. Data on sociodemographic, clinical and laboratory characteristics were extracted using a coded questionnaire, which in turn was entered into an excel sheet.

### Variables

Sociodemographic variables included age, sex, marital status, level of education and occupation.

Clinical variables included symptom duration prior to diagnosis, determined by diagnosis of CML less than or more than 12 months after onset of symptoms. Presence of B symptoms was defined as unintentional weight loss of ≥10 kg in the preceding 6 months, fevers, and night sweats. Time to development of cytopenia was defined as less than 3 months, 3–6 months and 6–12 months after imatinib initiation. Laboratory characteristics included the CBC with grade of cytopenia determined as per National Cancer Institute Common Terminology Criteria for Adverse Events v.3 [17]. Baseline BCR-ABL1 from RT-PCR was collected. We excluded the Sokal score risk as a variable since the percentage of bone marrow and peripheral blood myeloblasts that was missing from the records of our patients was high.

### Data Management

Data from excel was imported into the statistical analysis software for data management and analysis. Continuous data was presented using means and respective standard deviations (SD). Counts and corresponding percentages were used for categorical variables such as gender of participants and cytopenia group. Bivariate comparisons such as comparisons of cytopenia versus no cytopenia was done using chi square or fishers’ exact tests for categorical variables as deemed appropriate. Univariable logistic regression analysis was employed for demographic, clinical and laboratory variables associated with cytopenia. The odds ratio (OR) and 95% Confidence Intervals was also reported. Stata package, version 15.1 was used during statistical analysis. There were some (50 out of 201 records) BCR-ABL1 reports that were missing. To mitigate for this during the regression modeling, a category for missing data was created to ensure that the multivariable model included all the observations as available for all the covariates. Tables were used to display results. All methods were carried out in accordance with relevant guidelines and regulations.

## Results

Data on types, grades and time course of cytopenia has been analyzed and published [16].

### Baseline Characteristics

Ninety four (94) patients with cytopenia and 107 controls were included. Females were 97 and males were 104 of the total number of patients, 42% (85) of the 211 were aged 36–50 years, 30% (61) between 18 and 35 years and 27% (55) > 50 years, 73.1% (147) were married, 75.6% (152) were employed and 47.8% (96) and 46.8% (94) had secondary and tertiary education respectively. Clinically, 70% (142) had symptoms for ≥12 months before diagnosis, and 78.6% (152) had B symptoms at diagnosis. BCR-ABL was 0–25% for 35.8% (72), 26–75% for 27.9% (56) and > 75% for 11.5% (23) from 151 results available, baseline Hb was < 8 g/dl in 11% (22), 8–10 g/dl in 36.7% (73) and > 10 g /dl in 52.3% (104). Baseline neutrophil was > 7.5 to > 100 × 10^9^/L in 68% (134) and < 1.5 × 10 ^9^/L in 22 patients while the rest had normal levels. Baseline platelets count > 450 × 10^9^/L was found in 23.5% (47) and < 150 × 10^9^/L in 11% (22). There was similar distribution among cytopenia and no cytopenia groups.

Sex, age, marital status, occupation and education level were similar between the cytopenia and no cytopenia groups, *p* values > 0.05.

### Type of cytopena

Sixty-six patients (63.6%) among the 94 studied had a monocytopenia. Anemia developed in 32 (34%), neutropenia in 26 (27.6%) and thrombocytopenia in 8 (8%) of the 94 patients. Among patients with bicytopenia, the most common type was anemia plus neutropenia in 12 (12.7%) patients, followed by neutropenia plus thrombocytopenia in 8 (8%) patients and anemia and thrombocytopenia in 3 (3%) patients. Pancytopenia developed in 5 patients among the 94 studied (Table 1).

**Table 1:** Grades of cytopenia among the 66 patients with monocytopenia, NCI CTCAE, v3

Variable	Grade 2	Grade 3	Grade 4
Anemia (g/dL) n (%)	8–10	6.5–8	< 6.5
	17 (53)	13 (40.6)	2 (6.4)
Neutropenia (mm^3^) n (%)	≥1000–1500	500–1000	< 500
	10 (38.5)	14 (53.8)	2 (7.7)
Thrombocytopenia (mm^3^) n (%)	50,000–75,000	25,000–50,000	< 25,000
	5 (62.5)	2 (25)	1 (12.5)
**Total (n, %)**	32 (48.5)	29 (43.9)	5 (7.6)

Time duration to diagnosis, and positive B symptoms were similar between the cytopenia between the cytopenia and no cytopenia group (Table 2).

**Table 2:** Time to diagnosis

	Cytopenia	No cytopenia	*P* value
Early (< 12 months)	36 (33.6)	23 (24.5)	0.154
Late (> 12 months	71 (66.4)	71 (75.5)	
Spleen size			
11–20 cm	83 (77.6)	78 (83)	0.632
> 20 cm	3 (2.8)	2 (2.1)	
Normal	21 (19.6)	14 (14.9)	
B symptoms present at diagnosis			
No	26 (24.3)	17 (18.1)	0.284
Yes	81 (75.7)	77 (81.9)	

There was a significant difference in baseline Hb, baseline neutrophils, and baseline platelets.

There was a significantly lower proportion of the participants with 0–25% baseline BCR-ABL1 among the no cytopenia than the cytopenia group, *p* = 0.034 (Table 3).

**Table 3:** Bivariate Analysis of Laboratory Characteristics

Variable	No Cytopenia n (%)	Cytopenia n (%)	***P*** value
BCR-ABL at baseline (%)			
0–25%	37 (34.6)	35 (37.2)	0.034*
26–75%	25 (23.4)	31 (33)	
76–125%	13 (12.1)	6 (6.4)	
> 125%	0 (0)	4 (4.3)	
Missing	32 (29.9)	18 (19.1)	
Baseline platelets (× 10^9^)			
< 150	4 (3.8)	18 (19.1)	< 0.001*
150–450	80 (75.5)	51 (54.3)	
451–999	22 (20.8)	20 (21.3)	
1000+	0 (0)	5 (5.3)	
Baseline neutrophils (× 10^9^)			
< 1.5	4 (3.9)	18 (19.1)	< 0.001*
1.5–7.5	27 (26.2)	14 (14.9)	
7.6–100	58 (56.3)	32 (34)	
> 100	14 (13.6)	30 (31.9)	
Baseline HB (g/dL)			
< 6.5	1 (1)	2 (2.1)	< 0.001*
6.5–7.9	0 (0)	19 (20.2)	
8–10	47 (44.8)	26 (27.7)	
> 10	57 54.3)	47 (50)	

### Logistic regression Analysis

Demographic characteristics were not significantly associated with cytopenia (Table 4).

**Table 4:** Logistic Regression Analysis, Socio-Demographic Characteristics

	Univariable	*P* value	Multivariable	*P* value
	OR (95% CI)		OR (95% CI)	
Age Category				
17–35 years	0.991 (0.513–1.913)	0.978	1.153 (0.507–2.62)	0.734
36–50 years	Reference		Reference	
> 50 years	0.683 (0.343–1.357)	0.276	0.815 (0.308–2.15)	0.681
Gender				
Female	0.828 (0.475–1.442)	0.504	1.468 (0.589–3.65)	0.41
Male	Reference		Reference	
Marital Status				
Married	Reference		Reference	
Single/divorced/widow	1.194 (0.639–2.229)	0.578	0.965 (0.39–2.385)	0.938
Employment Status				
Not Employed	0.824 (0.402–1.689)	0.597	0.56 (0.195–1.611)	0.282
Employed	Reference		Reference	
Self Employed	0.42 (0.218–0.807)	0.009	0.36 (0.13–1.003)	0.051
Education Level				
Primary/Secondary	0.667 (0.382–1.164)	0.154	0.615 (0.25–1.511)	0.289
Tertiary	Reference		Reference	

Clinical characteristics were not significantly associated with the development of cytopenia (Table 5).

**Table 5:** Logistic Regression Analysis, Clinical Characteristics

	Univariable		Multivariable	
	Odds Ratio (95% CI)	*P* value	Odds Ratio (95% CI)	*P* value
Time duration to diagnosis				
Early	0.639 (0.344–1.185)	0.155	0.681 (0.154–3.016)	0.613
Late	Reference		Reference	
Spleen size				
Abnormal	Reference		Reference	
Normal	0.717 (0.341–1.505)	0.379	0.469 (0.122–1.794)	0.268
B symptoms				
No	0.688 (0.346–1.366)	0.285	1.368 (0.328–5.707)	0.667
Yes	Reference		Reference	

A baseline Hb < 7.9 g/dL, a baseline neutropenia < 1.5 × 10^9^/L and baseline platelet count > 450 × 10^9^/L or less than 150 × 10^9^/L were associated with increased odds of cytopenia in both univariable and multivariable analysis. Neutrophil counts above 100 × 10^9^/L increased the odds of cytopenia in the univariable analysis (Table 6).

**Table 6:** Logistic Regression Analysis, Baseline Laboratory Characteristics

	Univariable		Multivariable	
	OR (95% CI)	*P* value	OR (95% CI)	*P* value
Baseline BCR-ABL1 (%)				
0–25	Reference		Reference	
26–75	1.311 (0.65–2.642)	0.449	1.059 (0.421–2.662)	0.904
> 75	0.813 (0.316–2.092)	0.668	0.688 (0.192–2.463)	0.565
Missing	0.595 (0.284–1.246)	0.168	0.316 (0.111–0.905)	0.032
Baseline Neutrophils ×10^9^				
< 1.5	8.679 (2.459–30.63)	0.001	17.571 (3.909–78.987)	< 0.001
1.5–7.5	Reference		Reference	
7.6–100	1.064 (0.489–2.313)	0.875	1.087 (0.398–2.971)	0.87
> 100	4.133 (1.672–10.22)	0.002	2.776 (0.798–9.653)	0.108
Baseline Hb (g/dL)				
< 7.9	25.47 (3.302–196.4)	0.002	32.231 (3.502–296.653)	0.002
8–10	0.671 (0.363–1.241)	0.204	0.598 (0.269–1.327)	0.206
> 10	Reference		Reference	
Baseline platelets × 10^9^				
< 150	7.059 (2.26–22.05)	0.001	17.036 (4.079–71.157)	< 0.001
150–450	Reference		Reference	
451+	1.783 (0.91–3.491)	0.092	2.771 (1.083–7.088)	0.033

## Discussion

Data on types, grades and time course of cytopenia has been analyzed and published [16].

This was a study of 201 patients, 94 with cytopenia and 107 with no cytopenia. The number of females and males enrolled in the study was similar at 97 and 104 respectively with good gender distribution between the cytopenia and the no cytopenia groups. Data from the USA have reported that more males than females are affected and more females survive the disease in comparison to males [6]. Forty two percent (42%) of the patients were aged between 36 and 50 years, 73% were married, 75% were employed and literacy levels were high. These statistics are in keeping with the Kenya Demographic Health Survey (KDHS) data that reported that the country has a predominantly young population, 54.6% are married, and employment levels are 60 and 80% for males and females respectively. In addition, levels of literacy were high at > 80% among participants [19]. In contrast, and with respect to age, data from the developed countries have reported that CML is a disease of the older population, with the SEER database reporting a median age of 66 years [6].

Clinically, 70% of the patients had symptoms for ≥12 months before diagnosis, 78.6% had positive B symptoms, 80% had a moderate splenomegaly and 40% had used imatinib for ≤3 months and 34.7% for 3–6 months respectively before the cytopenia developed. The delay in diagnosis as evidenced by time to diagnosis and presence of B symptoms could be a result of weak health systems in Low- and Middle-Income Countries (LMIC) [20]. Likewise the findings of baseline neutrophilia in majority of our patients is a reflection of diagnosis of CML at an advanced stage. In contrast, in the developed countries, up to 50% are asymptomatic at diagnosis and when symptoms are present, splenomegaly is seen in 46–76% [21, 22].

Sex, age, marital status, occupation and education level were similar between the cytopenia and no cytopenia groups and they did not increase the odds of developing cytopenia. A study carried out in Iraqi reported that females on imatinib had a predilection for anemia compared to males [23]. This higher likelihood is probably due to the lower level of hemoglobin found in females at baseline compared to males [24]. Anemia may also be related to other comorbidities which might be confounding factors in the analysis [25].

Clinical symptoms such as time duration to diagnosis, spleen size and positive B symptoms are markers of advanced disease and were similar between the cytopenia group and the no cytopenia group. These factors did not however, increase the odds of cytopenia. Splenomegaly is a marker of advanced disease and is known to be significantly linked to poor outcomes [26]. Cytopenia has been reported to be more common in patients with advanced disease.

Blood counts and BCR-ABL1 differed significantly between the cytopenia and no cytopenia groups with more anemia, neutrophilia, thrombocytosis and thrombocytopenia in the cytopenia than the no cytopenia groups. Baseline anemia is known to accompany a higher baseline white blood cell counts, more frequent splenomegaly, and more CML related deaths [27]. Further, the anemia, neutropenia, thrombocytosis and thrombocytopenia increased the odds of cytopenia. Myelosuppression has been reported to contribute to poor response to treatment and to poor survival [28]. A study among 527 patients in Nigeria reported that baseline anemia was an independent prognostic factor for poor overall survival [29]. This is in contrast to a study conducted in Germany among CML patients that reported that hemoglobin level had no significant influence on overall survival [30]. The baseline cytopenia may be due to bone marrow dysfunction or fibrosis with fibrosis developing due to late presentation of CML [31]. Cytopenia persisting during treatment may also reflect disease progression or persistent disease [32]. Such patients should be closely monitored with additional bone marrow and molecular assays to assess response. However, cytopenia is rarely an indication for permanent discontinuation of imatinib. This is because majority of patients presenting with cytopenia show good marrow morphologic responses. However, sustained grade 2 cytopenia can be more important than occasional grade 3 and even if this, as is the case in our study, does not necessitate any clinical decision to pause therapy or reduce treatment dose, they do indeed require monitoring closely [8].

A few studies have reported that higher BCR-ABL1 levels are associated with poor outcomes. One study demonstrated that patients who experienced very severe myelotoxicity had a significantly higher BCR-ABL1 value after conducting FISH studies [33]. In our study, level of baseline BCR-ABL1 did not have any impact on hematological toxicity.

The study had a few limitations. Being retrospective, there was missing data that could have affected the outcomes of our study. Further, the study results can only be applied to patients with grade 2–4 cytopenia. It was not possible to risk score the patients fully and correlate prognostic score with cytopenia development due to inadequate numbers of patients with percent myeloblast reports. The Sokal and Hasford (Euro) prognostic scoring systems have been reported to predict outcomes such as overall survival in imatinib treated patients, such that patients with a high Sokal score had a lower OS at 10 years than those with intermediate or low scores [34].

However, the role of the risk scores in predicting development of cytopenia remains controversial.

High-risk patients who develop recurrent cytopenias on treatment may be more prone to develop aplasia. But, other risk scoring tools besides sokal have not definitively shown a correlation between high risk disease and development of myelosuppression, implying that myelosuppression is not merely a reflection of more advanced disease. Conversely, whether intermediate or low risk score patients have lower risk of developing myelosuppression remains a subject of active investigation.

## Conclusions

Our findings are similar to those of studies conducted both in sub-Saharan Africa and in the developed world. We recommend that physicians should have a high index of suspicion to recognize patients at risk of developing cytopenia. This includes patients with low baseline cytopenia as well as patients with baseline thrombocytosis.

## Supplementary Information

**Additional file 1.**

## Data Availability

The dataset(s) supporting the conclusions of this article is (are) included within the article (and its additional file(s) which can be provided).

## Abbreviations

CML: Chronic Myeloid Leukemia
GIPAP: Glivec International Patient Access Program
KNH/UON: Kenyatta National Hospital/University of Nairobi
KDHS: Kenya Demographic Health Survey
LMIC: Lower- and Middle-Income Countries
TKI: Tyrosine Kinase Inhibitor
